# Understanding admixture patterns in supplemented populations: a case study combining molecular analyses and temporally explicit simulations in Atlantic salmon

**DOI:** 10.1111/j.1752-4571.2012.00280.x

**Published:** 2012-06-14

**Authors:** Charles Perrier, Jean-Luc Baglinière, Guillaume Evanno

**Affiliations:** 1 INRA, UMR 0985 Ecology and Health of EcosystemsRennes, France; 2 Agrocampus OuestRennes, France; 3 INRA, UMR 1313 Animal Genetics and Integrative Biology, Domaine de VilvertJouy-en-Josas, France; 4 Département de Biologie, Institut de Biologie Intégrative et des Systèmes, Université Laval, QuébecQC, Canada

**Keywords:** admixture, conservation, *Salmo salar*, simulation, stocking

## Abstract

Genetic admixture between wild and introduced populations is a rising concern for the management of endangered species. Here, we use a dual approach based on molecular analyses of samples collected before and after hatchery fish introduction in combination with a simulation study to obtain insight into the mechanisms of admixture in wild populations. Using 17 microsatellites, we genotyped pre- and post-stocking samples from four Atlantic salmon populations supplemented with non-native fish to estimate genetic admixture. We also used individual-based temporally explicit simulations based on realistic demographic and stocking data to predict the extent of admixture. We found a low admixture by hatchery stocks within prestocking samples but moderate to high values in post-stocking samples (from 12% to 60%). The simulation scenarios best fitting the real data suggested a 10–25 times lower survival of stocked fish relative to wild individuals. Simulations also suggested relatively high dispersal rates of stocked and wild fish, which may explain some high levels of admixture in weakly stocked populations and the persistence of indigenous genotypes in heavily stocked populations. This study overall demonstrates that combining genetic analyses with simulations can significantly improve the understanding of admixture mechanisms in wild populations.

## Introduction

Genetic admixture between wild indigenous populations and introduced conspecifics from distant and/or captive stocks is a growing concern in conservation biology (Allendorf et al. 2001; Lecis et al. 2006; Randi 2008; Hansen and Mensberg 2009). Wild Salmonid populations are especially affected by the introduction of hatchery-reared individuals and by escapees from fish farms (Levin et al. 2001; Aprahamian et al. 2003; Fraser 2008; McGinnity et al. 2009). Because captive breeding may select for traits that are disadvantageous in the wild (Blanchet et al. 2008; Fraser 2008; Williams and Hoffman 2009) and because salmonid populations are often locally adapted (Garcia de Leaniz et al. 2007; Fraser et al. 2011), admixture between wild and introduced salmonids may ultimately result in a loss of local adaptation and a fitness reduction in wild populations (McGinnity et al. 2003; Araki et al. 2007a; Ford and Myers 2008; but see Fitzpatrick et al. 2010). Therefore, estimating the admixture between introduced and wild populations is a major challenge to preserve the genetic diversity of wild populations and to ameliorate supplementation strategies (Randi 2008; Sonstebo et al. [Bibr b57]; Hansen et al. 2009; Winkler et al. 2011).

Salmonid populations are genetically structured throughout their native range (Verspoor et al. 2005; Lehtonen et al. 2009; Tonteri et al. 2009) and admixture between stocks is generally inferred using mtDNA (Campos et al. 2008), microsatellites (Finnengan and Stevens 2008; Hansen et al. 2009) or SNPs (Bourret et al. 2011). Furthermore, the temporal evolution of population genetic structure can be investigated using time series of archived scales (Nielsen and Hansen 2008; Quinn and Seamons 2009). Several studies reported variable admixture rates depending on stocking intensity in Atlantic salmon (Campos et al. 2008; Finnengan and Stevens 2008), brown trout (Sonstebo et al. [Bibr b57]; Hansen et al. 2009), and brook charr (Marie et al. 2010). However, admixture rates may not only depend on stocking intensity but also on local population size (Currat et al. 2008; Hansen et al. 2009), survival and reproductive success of hatchery fish in the wild (Araki et al. 2008) and dispersal of both wild and stocked fish (Perrier et al. 2011a). Given this high number of factors involved in the admixture process, comparative analyses of observed admixture levels with simulated admixture data based on realistic demographic parameters may give some insight into the mechanisms of admixture in wild populations. Such an approach may allow predicting admixture levels before supplementation operations and thus significantly improves management strategies.

Several individual-based simulation methods are available to study the temporal evolution of population genetic structure (e.g., the softwares Easypop (Balloux 2001), Splatch (Currat et al. 2004), Nemo (Guillaume and Rougemont 2006), and Hybridlab (Nielsen et al. 2006)). Some of them were successfully used to simulate genetic introgression following demographic expansions or bioinvasions (Currat et al. 2008). In the case of human-mediated interpopulation transfers, local population sizes and the number of non-native individuals transferred are generally known. As a result, it can be possible to compare observed admixture levels with expected levels simulated according to quantitative data available on transfers of individuals. For instance, Hansen (2002) compared the introgression computed with simulations to the one estimated from microsatellite analyses in a brown trout population. Simulations were based on the assumption of equal survival and reproductive performances among hatchery and wild fish. He observed a much lower genetic contribution of hatchery fish to the wild population than expected with simulated data, suggesting poor performances of hatchery fish. Recent simulation tools (reviewed in Hoban et al. 2012) allow modeling more realistic scenarios where the effect of variations in dispersal and/or survival can be tested in a metapopulation framework (see for instance the Nemo program by Guillaume and Rougemont 2006).

Here, we assessed the admixture between wild and hatchery fish in four Atlantic salmon (*Salmo salar*) populations from the Baie-du-Mont-Saint-Michel (BMS), Lower-Normandy, France, using microsatellite DNA analyses of pre- and post-stocking samples. From 1989 to 2003, about 1 052 000 young-of-the-year salmon have been introduced in the Couesnon (COU), Sélune (SEL), Sée (SEE), and Sienne (SIE) rivers. Individuals released were non-native and produced by progenitors captured in distant French populations: the Aulne River (Brittany) and to a lesser extent the Gave d'Oloron River (Aquitania). Scale samples of fish caught by anglers have been collected since the late 1960s in BMS populations. In addition, estimates of population sizes are available and the numbers and origins of fish stocked in each river are known. We used available demographic, genetic, and stocking data to run individual-based simulations to produce expected admixture rates according to various demographic scenarios. Specifically, this study aimed at (i) comparing the genetic structure and admixture levels of the study populations before and after stocking, (ii) simulating realistic expected admixture rates in each population according to stocking and demographic data, and (iii) comparing the observed data with simulated admixture data to determine whether lower survival and higher dispersal of stocked fish relative to wild individuals may explain the observed patterns of admixture.

## Material and methods

### Study populations and sampling

**Figure 1:**
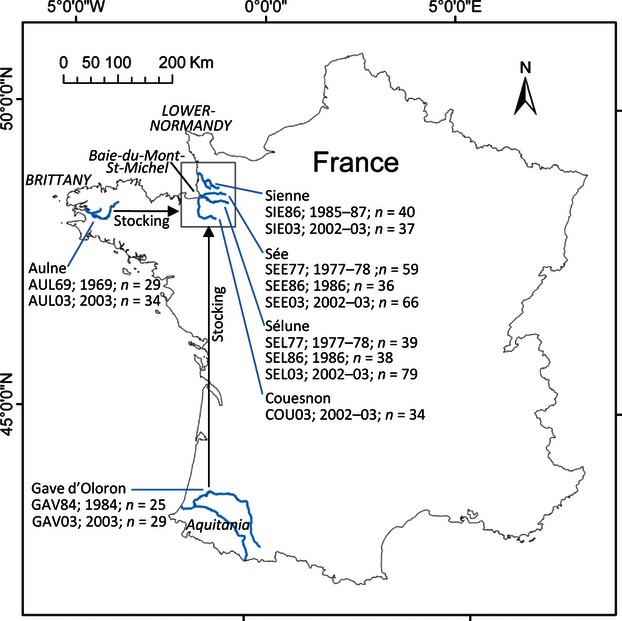
Map of study rivers with sample sizes in each site. Aulne and Gave d'Oloron populations have been used to stock Couesnon, Sélune, Sée, and Sienne rivers.

The location of the four study populations (COU, SEL, SEE, and SIE) is presented in Fig. 1. These populations declined in the 1950s probably due to important habitat degradations. COU population was considered extinct (no more natural reproduction) in the 1970s. As a result, supplementation operations have started in 1989 using non-native young-of-the-year (0+) produced in the Favot Hatchery, Brittany. Spawners caught every year in the Aulne River (AUL, Fig. 1) were used to produce juveniles that were stocked in autumn and winter (i.e., at 9–12 months). The four BMS rivers were stocked with variable number of individuals, as indicated in Table 1. From 1989 to 1994 and from 1996 to 2003, approximately 931 000 fish produced by AUL progenitors were stocked (Table 1). In 1995, no fish originating from AUL were stocked but instead about 80 000 juveniles originating from progenitors caught in the Gave d'Oloron River (GAV) were released. COU has been stocked from 1989 to 2003 with a total of 623 518 fish. SEL has been stocked from 1989 to 1996 with a total of 363 500 fish. SEE and SIE were stocked once in 1990 with 14 000 and 10 000 individuals, respectively.

**Table 1:** Demographic and stocking data for the Couesnon, Sélune, Sée, and Sienne populations from 1989 to 2009. Stocked fish originated from Aulne except in 1995 where individuals from Gave d'Oloron were used (Given in bold). Estimates of adult population sizes and average 0+ autumn parr productions are indicated

	Average population size	Average 0+ parr production	Stocking																				

			1989	1990	1991	1992	1993	1994	1995	1996	1997	1998	1999	2000	2001	2002	2003	2004	2005	2006	2007	2008	2009
COU	200	4000	22 000	53 500	25 000	64 500	25 000	16 600	**50 267**	43 197	89 020	59 665	48 200	30 782	35 049	33 172	27 566	29 381	23 585	22 988	25 519	20 090	27 094
SEL	300	6000	4000	36 000	25 900	30 000	101 000	66 000	**29 800**	61 000	9800	–	–	–	–	–	–	–	–	–	–	–	–
SEE	800	16 000	–	14 000	–	–	–	–	–	–	–	–	–	–	–	–	–	–	–	–	–	–	–
SIE	400	8000	–	10 000	–	–	–	–	–	–	–	–	–	–	–	–	–	–	–	–	–	–	–

All samples were scales collected on anadromous adults caught by anglers and stored by INRA (*Institut National de Recherche Agronomique*) and ONEMA (*Office National de l'Eau et des Milieux Aquatiques*). The age of each individual was determined from its scales. As post-stocking samples, we focused on cohorts 2002–2003 (Fig. 1, Table S1). For prestocking samples, we chose cohorts 1977–1978 (SEE and SEL) and 1985 to 1987 (SIE, SEE and SEL). For the two populations where progenitors were collected for the hatchery, we chose samples from cohorts 1969 and 2003 for AUL, and 1984 and 2003 for GAV. A total of 545 samples (from 25 to 79 individuals per temporal sample) were genotyped.

### Molecular analyses

Genomic DNA was extracted from *S. salar* scales by heating samples in a solution of proteinase K, TE (Tris/EDTA) buffer, and chelex, at 55°C 2 h and then at 100°C for 10 min (Estoup et al. 1996). Individuals were genotyped at 17 microsatellites (BHMS176; BHMS179A; BHMS184B; BHMS429; SSA85; SSA65; SSOSL85; SSA9; BHMS235; BHMS217; BHMS111; SSA197; SSA171; BHMS377; SSSP2216; SSA224; BHMS365) following the procedure described in Perrier et al. (2010).

### Molecular data analyses

We used Micro-Checker 2.2.3 (Van Oosterhout et al. 2004) to assess the frequency of null alleles and scoring errors attributed to stuttering or large allelic dropout. Allele number and allelic richness were obtained using Fstat 2.9.3.2 (Goudet 1995). Tests for linkage and Hardy–Weinberg Equilibrium (*via F*_IS_) were also conducted with Fstat. Expected heterozygosity, He, (Nei 1978) and observed heterozygosity, Ho, were calculated with Genetix 4.05.2 (Belkhir et al. 1996). Fdist 2 (Beaumont and Nichols 1996) was used to verify the neutrality of the markers used. Pairwise *F*_ST_ and tests of differentiation were conducted in Fstat. Pairwise Nei (Da) genetic distances (Nei et al. 1983) were estimated using Populations 1.2.30 (http://bioinformatics.org/∼tryphon/populations/).

As per the studies of Hansen and Mensberg (2009), Marie et al. (2010), and Winkler et al. (2011), admixture between wild and hatchery stocks was estimated at the individual level using the Bayesian method implemented in the Structure software (Pritchard et al. 2000). Structure analyses were performed assuming an admixture model with default settings (i.e., no informative prior was used). We ran Structure from 1 to 6 genetic clusters (*k*) with 15 replicates for each *k*. Each run started with a burn-in period of 50 000 steps followed by 300 000 Markov Chain Monte Carlo replicates and estimating 90% credible intervals. We selected the *k* with the highest likelihood (Pritchard et al. 2000) and according to the Δ*k* method (Evanno et al. 2005). We then calculated average population admixtures at the best clustering solution. We also estimated an overall admixture in the four BMS populations calculated as the sum of the admixture of each population weighted by its size (number of returning adults).

### Simulation study

We used Nemo 2.1.0, a stochastic individual-based genetically explicit framework (Guillaume and Rougemont 2006) to simulate the evolution of a metapopulation made of wild and hatchery individuals. Our aim was to produce expected patterns of admixture based on various demographic scenarios, which could be compared with the observed data. An individual-based approach was used to produce data as similar as possible to molecular data.

Modeled organisms were diploid with separate genders and lived in a structured metapopulation of six demes with the following local carrying capacities: 2000, 7000, 200, 300, 800, and 400 adults, corresponding to the GAV, AUL, COU, SEL, SEE, and SIE populations, respectively (see also Table 1 and Fig. S1). These carrying capacities were assumed to correspond to the population sizes of COU, SEL, SEE, and SIE estimated from average 0+ parr abundance data (Anonymous 2008). These data were collected by local freshwater fishery organizations through annual electrofishing campaigns between 2001 and 2008. To estimate adult population sizes, we first applied a survival rate of 50.3% between 0+ parr and smolt stages estimated by Baglinière et al. (2005) over the 1995–2003 period in the Oir River, a tributary of the SEL River. Second, we applied a 9.67% survival rate between smolt and adult stages, which was estimated by Etienne Prévost (unpublished data) in the Scorff River (French index river for International Council for the Exploration of the Sea) over the 1995–2008 period. The final estimates were rounded to the nearest ten (Table 1). For GAV and AUL, we chose artificially high population sizes to allow high dispersal rates simulating stocking operations. To assess the sensitivity of our approach to varying population sizes, we also implemented eight additional values in BMS populations for one of the most probable scenarios (C7, see Table 2). The four initial population sizes were multiplied by 0.5, 0.7, 0.8, 0.9, 1.1, 1.2, 1.3, and 1.5 (Table 2).

**Table 2:** Description of combinations of parameters used for the different simulated scenarios. Survival rates are indicated as ratios between stocked and wild fish (the coefficient by which the survival of stocked fish was divided relative to wild individuals is given in brackets). Pairs of dispersal rates are given for wild and stocked individuals. Different mating systems and population sizes were further investigated for one of the most probable scenarios (C7)

Survival rate of stocked fish relative to wild individuals	Dispersal of wild; stocked fish			

	0.06; 0.06	0.06; 0.15	0.15; 0.15	0.15; 0.24
1.00 (1)	A1	B1	C1	D1
0.10 (10)	A2	B2	C2	D2
0.09 (11)	A3	B3	C3	D3
0.08 (12)	A4	B4	C4	D4
0.07 (14)	A5	B5	C5	D5
0.06 (16)	A6	B6	C6	D6
0.05 (20)	A7	B7	C7*^,^†	D7
0.04 (25)	A8	B8	C8	D8
0.03 (33)	A9	B9	C9	D9
0.02 (50)	A10	B10	C10	D10
0.01 (100)	A11	B11	C11	D11

* In addition to monogamy, 20% and 50% of polygamy were tested for this scenario.

† Varying population sizes (initial sizes multiplied by 0.50, 0.70, 0.80, 0.90, 1.10, 1.20, 1.30, and 1.50) were tested for this scenario.

We implemented the following semelparous life cycle: (i) breed; (ii) dispersal; (iii) random regulation of local populations size, which reduced the pool of competing individuals to the local carrying capacity (with equal sex ratios); (iv) reproduction during which females were assigned a fecundity value drawn from a Poisson distribution with a mean value of 40 offspring and mated with one randomly chosen male (monogamy). We also investigated the potential effect of multiple mating by testing 20% and 50% of polyandry for one of the best scenarios. Adults died after reproduction and the cycle started again. This assumption of semelparity is realistic as the estimated proportion of multispawners in BMS salmon populations is extremely low: from 0.9% to 2.1% for the 1992–2002 and 1972–1982 periods, respectively (Baglinière et al. 2004). We simulated 17 unlinked neutral loci with initially 15 alleles per locus, a mutation rate *u* = 0.0001, and a recombination rate *U* = 0.5. Alleles were inherited randomly (i.e., no linkage or epistasis) and sex was set randomly (equal sex ratio). *F*-statistics and multilocus genotypes were recorded every generation. There was no dispersal between AUL, GAV, and the four other BMS populations before stocking events.

Using dispersal values reported in the literature for Atlantic salmon (Jonsson et al. 2003a; Pedersen et al. 2007), we implemented four pairs of dispersal rates for wild and stocked fish among BMS populations: 0.06/0.06, 0.06/0.15, 0.15/0.15, and 0.15/0.24, respectively (Table 2). These values correspond to the total dispersal from a given population with, for example, a rate of 0.06 meaning a 0.02 fraction of migrants from a river move into each of the three others.

We investigated the effects of a lower survival of hatchery fish compared with the wild individuals by testing 11 hatchery/wild ratios of survival, from 1 to 0.01 (Table 2). Dispersal rates from GAV and AUL to BMS populations were modified at each generation to simulate the evolution of stocking practices. We simulated 44 scenarios with varying combinations of survival and dispersal rates, and for one of the most probable scenarios, we tested the sensitivity of our approach to variations in population size and mating system (Table 2). Each configuration was run three times for 290 generations to reach equilibrium and obtain a genetic structure similar to the observed prestocking situation. Then, we implemented stocking events from the generation 291 on according to the stocking data and we ran the program until generation 296. In BMS salmon populations, most juveniles spend 1 year in freshwater and most adults spend 1 year at sea and spawn at the end of their third year (Baglinière et al. 2005). Given this predominant generation time of 3 years, we modified the dispersal matrix at each generation according to the stocking data averaged over three consecutive years (generation 291: 1989–1991; g292: 1992–1994; g293: 1995–1997; g294: 1998–2000; and g295: 2001–2003). We used adults born at the generation 295 for admixture analyses.

Three Structure runs were conducted for each Nemo run at the best clustering partition (k=3) with the same settings used for molecular data. To estimate population admixtures, we averaged admixture values over the three Structure runs and then averaged values of the three Nemo runs. To identify the simulated scenario(s) that best fitted the observed data, we used two approaches. First, we used chi-square tests to test whether mean-simulated admixture values for each scenario significantly differed from observed values. Second, we used a least-squares approach: we summed the squared differences between observed and simulated individual admixture values for each scenario in each population and defined the best scenario as the one with the smallest sum of squared differences. The best scenario for the situation where admixture rates were averaged over the four populations was defined as the smallest of the four sums of squares, which were divided by the corresponding number of individuals in each population. We also used factorial correspondence analyses computed with Genetix 4.05.2 to display the genetic structure among samples and compare observed and simulated data.

## Results

### Genetic variation within populations

The average successful amplification and scoring rate per locus was 97.5% for contemporary samples and 92.3% for historical ones with no difference between loci or populations. No significant genetic differentiation (*F*_ST_) was found between successive cohorts in SIE, SEE, and SEL, indicating temporal stability of allele frequencies within populations. We thus combined genotypes from successive cohorts for each population in our analyses. We found some evidence of null alleles or large allele dropouts for six of 238 tests. Considering the overall data set, there was no evidence of departure from HWE (Table S2) nor LD associated with a particular marker, thus ruling out locus-specific factors. Average gene diversity (He) over all populations was 0.77, ranging from 0.74 to 0.80.

### Genetic variation among populations

**Figure 2:**
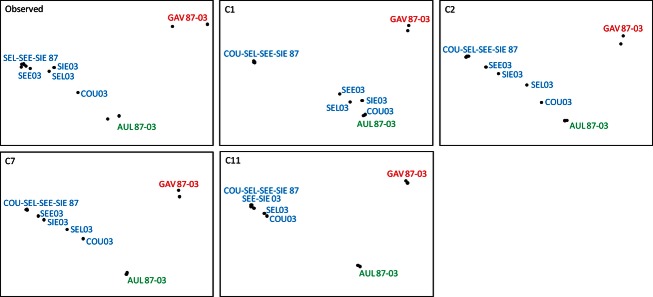
Factorial correspondence analyses showing genetic distances between samples for observed and simulated data (scenarios C1, C2, C7, and C11).

Pairwise tests of genetic differentiation yielded significant outcomes among GAV, AUL, and BMS populations for both historical and contemporary samples (Table S3). There was no significant differentiation among historical samples from SEL, SEE, and SIE as well as among contemporary samples (*F*_ST_ < 0.01). In contrast, *F*_STs_ between COU03 and the other BMS samples were significant (0.01–0.03). *F*_STs_ among temporal samples were relatively low but significant for SIE86-SIE03, SEL86-SEL03, and AUL69-AUL03 (Table S3). The differentiation between BMS populations and GAV and AUL was slightly higher among historical samples (*F*_ST_ = 0.08; from 0.057 to 0.096) than among contemporary samples (*F*_ST_ = 0.04; from 0.013 to 0.071, Table S3). In particular, COU03 was less differentiated from GAV03 and AUL03 than SEL03, SEE03, and SIE03. The factorial correspondence analysis highlighted the existence of three genetic groups corresponding to populations from BMS, AUL, and GAV (Fig. 2). This analysis also revealed lower genetic distances between contemporary BMS samples than old ones (Fig. 2).

### Admixture within populations

**Figure 3:**
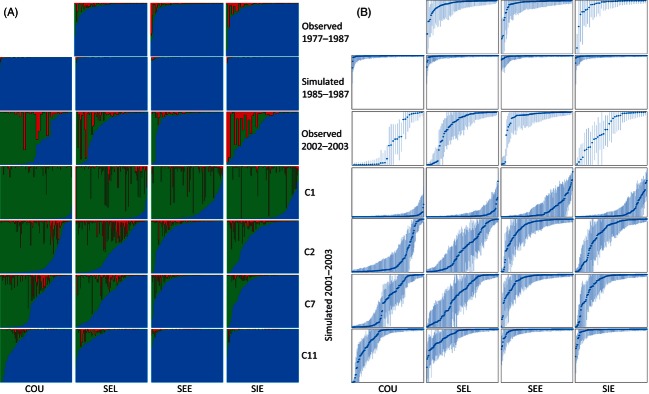
(A) Individual admixture proportions between the Gave d'Oloron (red bar), Aulne (green bar), and Normandy (blue bar) clusters in the four BMS populations (COU, SEL, SEE, and SIE) for observed and simulated data. Vertical bars show the proportions of individual membership to each cluster. (B) Individual admixture proportions of the local cluster (Normandy) with 90% credible intervals. Admixture analyses of simulated data are shown for four scenarios with 150 randomly sampled genotypes per population.

The Structure program consistently identified three genetic clusters (*k*) corresponding to AUL, GAV, and BMS samples (Fig. 3). 90% credible intervals of admixture values were relatively narrow in most cases. Admixture proportions from AUL and GAV clusters in historical samples of BMS were small: from 0.02 to 0.05. Admixture proportions of AUL and GAV were of same magnitude in SEL77, SEL86, SEE77, and SEE86. In contrast, we found a high genetic contribution of the AUL cluster in post-stocking samples of COU03, SEL03, SEE03, and SIE03: 0.53, 0.20, 0.09, and 0.16, respectively (Table 3). The GAV cluster contribution in COU03, SEL03, SEE03, and SIE03 was also higher than in old samples but remained relatively small: 0.07, 0.08, 0.03, and 0.14, respectively. Considering these four BMS populations and their relative sizes, the overall weighted admixture of BMS with AUL and GAV was 0.25. A close inspection of admixture in COU03 revealed many individuals with high proportions of the AUL cluster (close to one) and few admixed individuals (Fig. 3). Contrastingly, in SEL03, SEE03, and SIE03, admixed individuals were more numerous than individuals with high memberships from AUL or GAV clusters.

**Table 3:** Average admixture (±standard deviation) between hatchery stocks (Gave d'Oloron and Aulne) and Couesnon, Sélune, Sée, and Sienne populations in cohorts 2002–2003 for observed and simulated data. For each simulated scenario, admixture values are presented for each population and over all BMS populations and for each source of admixture [AUL, Gave d'Oloron River (GAV), and both together]. Simulated values that did not significantly differ from observed data are indicated in grey (chi-square test; *P*-value > 0.05) and the best scenario within each dispersal group (A, B, C, and D) according to the least-squares approach is indicated in bold. Overall weighted admixtures were calculated as the sum of admixtures of each population weighted by its average size

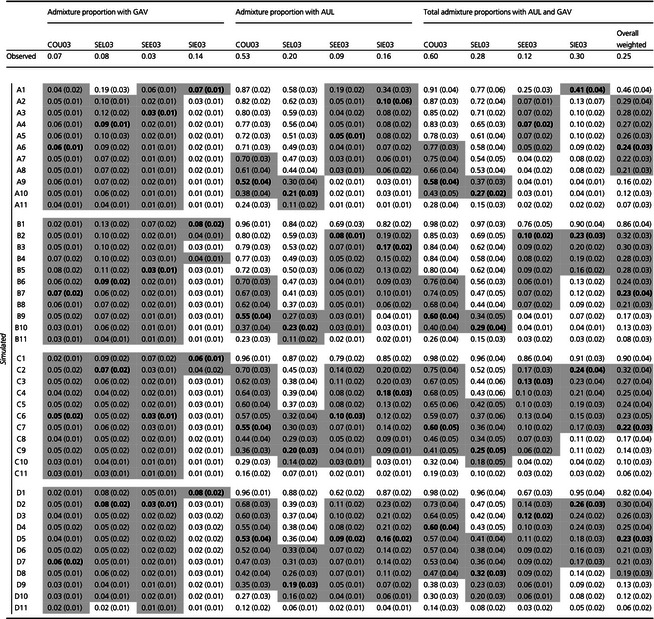

### Genetic variation among simulated populations

Pairwise tests of genetic differentiation yielded significant outcomes among GAV, AUL, and BMS populations for pre stocking data and most of post-stocking samples (data not shown). The differentiation between post-stocking BMS samples and GAV and AUL was comparable to the one observed in real data (average *F*_ST_ = 0.07; from 0.058 to 0.077). Similarly, the differentiation among prestocking samples from BMS was low in all scenarios (*F*_ST_ < 0.01). The factorial correspondence analysis highlighted the existence of three genetic groups among simulated populations before stocking (Fig. 2). In the scenario C1 where stocked and wild fish had the same survival, all contemporary BMS samples were close to AUL. The scenarios C2 and C7 revealed a gradient of differentiation between pre- and post-stocking samples of SEE, SIE, SEL, and COU. In the scenario C11 with the lowest survival of stocked fish, the genetic characteristics of BMS populations remain mostly unchanged. Overall, among these four scenarios, the C7 best fitted the observed data.

### Admixture within simulated populations

**Figure 4:**
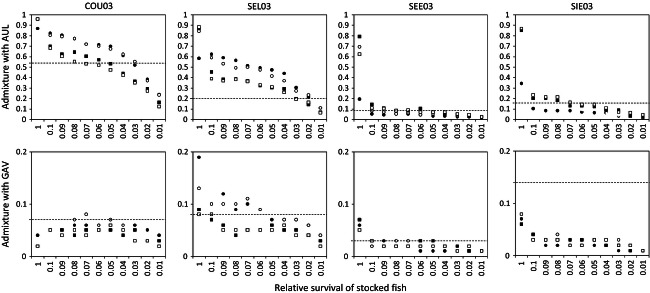
Simulated population admixtures with Aulne and Gave d'Oloron in BMS populations as a function of varying relative survival rates of stocked fish. Filled circles, open circles, filled squares, and open squares indicate dispersal rates of wild and stocked fish implemented in scenarios A, B, C, and D, respectively (see Table 2). Dotted lines indicate observed admixture levels with Aulne and Gave d'Oloron.

In simulated data, the Structure program identified three genetic clusters (*k*) corresponding to AUL, GAV, and BMS samples (Table 3 & Fig. 3). The variability of admixture estimates among Nemo and Structure runs was relatively low for a same scenario (average standard deviations of 0.03 and 0.01, respectively). 90% credible intervals were relatively narrow and of the same magnitude as for molecular data (Fig. 3). Admixture proportions of AUL and GAV clusters in BMS populations were small before stocking events: 0.01 on average. After stocking, the admixture in COU03, SEL03, SEE03, and SIE03 by GAV was low in most cases (Table 3). Chi-square tests revealed no significant differences between simulated and observed admixture by GAV for most scenarios in COU03, SEL03, and SEE03. In contrast, in SIE03 the observed admixture by GAV was in most cases significantly higher than simulated values. We found a wide range of admixtures by AUL in BMS populations: from 0.01 to 0.96 depending on the scenarios (Table 3, Figs 3 and 4). The majority of simulated scenarios were not significantly different from observed admixture levels by AUL but importantly all scenarios implementing an equal survival of stocked and wild fish significantly differed from the observed data. For this last category of scenarios, simulated admixture was much higher than observed admixture (Table 3). Scenarios implementing a 100 times lower survival of stocked fish produced significantly lower admixture values by AUL than the observed data.

When considering the total admixture by AUL and GAV, a high number of scenarios did not significantly differ from the observed data, but all but one of the scenarios with equal survival of wild and stocked fish produced significantly higher admixture levels than the observed data (Table 3). Among the scenarios considering overall admixture weighted by population size, those implementing a 10–25 times lower survival for stocked fish, were not significantly different from observed admixture levels. Data presented in Fig. 4 confirm that this pattern holds in most cases except for the admixture of SIE3 by GAV. In this population, simulated scenarios always predict admixture levels by GAV much lower than the one observed whatever the survival or dispersal rates tested.

The four pairs of dispersal rates tested (A, B, C, and D) produced similar number of scenarios not differing from the observed data when considering global levels of admixture (Table 3). At the population level, scenarios with higher dispersal rates (Cs and Ds) were more similar to the observed data from COU03, SEL03, and SIE03. However, for SEE03 most dispersal rates tested produced admixture levels not different from the observed data (Table 3). Data from Table 3 also reveal that dispersal was globally negatively linked to simulated admixture in highly stocked populations (COU03 and SEL03). Conversely, dispersal was positively related to admixture in weakly stocked populations (SEE03 and SIE03).

Overall, admixture proportions for the scenario C7 fitted well with the observed data (Table 3). This scenario was thus used to explore the sensitivity of simulation data to variations in population size and mating system (Table 4). Results only slightly varied when we implemented a 20% and 50% polyandry mating systems (Table 4). However, significant differences appeared with varying population sizes: admixture increased when population sizes decreased (Table 4). Conversely, simulated admixture rates only slightly decreased when population sizes were increased from 10% to 50%.

**Table 4:** Effect of variations in mating system and population sizes on average admixtures with hatchery stocks (Gave d'Oloron and Aulne) in BMS populations for one of the most probable simulated scenario (C7). Standard deviations among nemo runs are indicated in brackets. Simulated admixture values that did not significantly differ from the observed data are indicated in grey (chi-square test; *P*-value > 0.05), and the best scenario according to the least-squares approach is indicated in bold

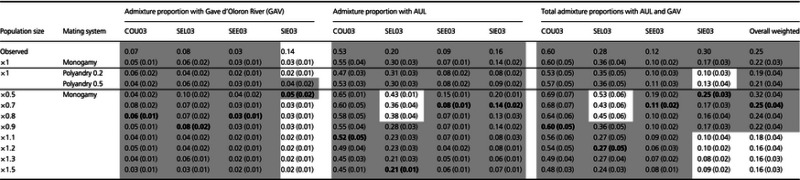

## Discussion

This study showed moderate to high admixture levels between wild and stocked salmon in post-stocking BMS samples. Importantly, a moderate level of admixture by Gave d'Oloron was observed in the SIE population while fish from the former had not been stocked in the latter but in other BMS populations. This demonstrates that stocked fish (or their wild-born descendants) can disperse into unstocked rivers and modify their genetic makeup. By comparing molecular data with realistic temporally explicit simulations, we showed that stocked salmon may have a 10–25 times lower survival than wild individuals. Simulations did not support the hypothesis of a higher dispersal of stocked fish relative to wild individuals, but this aspect requires further investigations.

We found significant admixture rates with Aulne and Gave in post-stocking samples from BMS (from 12% to 60%) while prestocking samples were weakly admixed (from 2% to 5%). Furthermore, our results showed a decrease in genetic differentiation among donor and stocked populations. Some of these BMS populations have been intensively stocked during the last decades and were the only ones in France to be still stocked with non-native fish in the 2000s. Therefore, while the weak admixture observed in prestocking samples most probably results from low levels of ‘natural’ gene flow, high contributions of donor populations within post-stocking samples are consistent with stocking data and corroborate previous results on these populations (Grandjean et al. 2009; Perrier et al. 2011a,b). These results are also consistent with studies showing a correlation between stocking intensities and admixture in Atlantic salmon (Campos et al. 2008; Finnengan and Stevens 2008), brown trout (Sonstebo et al. [Bibr b57]; Hansen et al. 2009), and brook charr (Marie et al. 2010).

The comparison between observed and simulated admixture data in BMS samples suggests a relatively low survival of stocked fish. However, this observation does not hold for fish originating from Gave d'Oloron because the admixture by this population was very low and almost all simulated scenarios were compatible with the observed data (except for the SIE population). This suggests that our simulation approach cannot be used to infer relative survival rates of stocked fish when admixture is very low (<10%). The overall admixture rate by Aulne and Gave d'Oloron in BMS populations was 25% (range 12–60%), which allowed meaningful comparisons with simulation scenarios. For instance, the overall observed admixture was always significantly different from simulated scenarios with an equal survival of wild and stocked fish, which predicted 46–90% of admixture. Scenarios compatible with overall observed admixture suggested a 10–25 times lower survival of stocked fish relative to wild individuals. At the population level, our simulations suggest that only 2–9% of the native gene pool may remain in COU if the survival of stocked and wild fish was equal, whereas we observed that 40% of the native gene pool remains. The level of admixture in the observed data could result from a low survival of hatchery fish in the wild (Jonsson et al. 2003b; Theriault et al. 2008) but also from a lower reproductive success (Araki et al. 2007a, 2009; Theriault et al. 2011). Such lower performances of stocked fish may be explained by a variety of genetic or environmental effects, but we cannot discriminate these effects with our data.

Most observed admixtures corresponded well to the scenarios implementing a low survival of stocked fish except in the SIE population where scenarios with an equal survival of hatchery Gave d'Oloron fish and wild individuals were compatible with the observed data. However, no fish from Gave d'Oloron were stocked in the Sienne river where 14% of admixture with the former was observed. Several nonexclusive hypotheses can explain this result. First, fish from Gave d'Oloron stocked into COU and SEL (or their wild-born descendants) must have dispersed into the Sienne River. Several studies have reported high straying rates of stocked salmonids (Quinn 1993; Jonsson et al. 2003a; Pedersen et al. 2007), which may have important consequence on the genetic structure of unstocked populations located near stocked rivers (Perrier et al. 2011a). Second, stocked fish could have been better adapted to local environmental conditions or have benefited from large areas of unoccupied salmon habitat at the time of stocking. Third, environmental stochasticity linked to a large variance of individual reproductive success, juvenile survival, and population size among years and/or rivers could also explain this result (Dumas and Prouzet 2003; Dannewitz et al. 2004; Araki et al. 2007b). Finally, the sensitivity analysis of our simulation approach to varying population sizes suggests that a lower size of the SIE population at the time of stocking may explain the higher success of stocked fish in this river.

Empirical data suggested a high dispersal of stocked fish leading to relatively high admixture rates in weakly stocked SIE and SEE populations. Accordingly, the high admixture with the local cluster observed in the previously almost extinct COU population suggests significant levels of dispersal of wild BMS individuals. Our simulation study suggested that both 6% and 15% dispersal rates were compatible with overall observed admixture data. However at the population level, 15% dispersal rates produced more scenarios compatible with the observed data in COU, SEL, and SIE populations, suggesting that high dispersal was an important factor in the establishment of high admixture rates in these populations. Nevertheless, simulated scenarios implementing a higher dispersal rate of stocked fish relative to wild individuals did not better fit the observed data than scenarios with equal dispersal rates for the two groups of individuals. Straying rates were chosen according to Jonsson et al. (2003a), but higher values up to 67% for hatchery-reared fish were also suggested in this study but have not been investigated in ours. In addition, the dispersal of individuals born in the wild from hatchery parents may further facilitate the propagation of hatchery genes among BMS rivers (Perrier et al. 2011b). An important straying of native BMS individuals may also explain the relatively low admixture of non-native clusters into the depopulated but stocked Couesnon River (Vasemagi et al. 2001; Perrier et al. 2010).

Despite the use of a restricted set of simulation parameters, we detected some combinations of parameters that fitted remarkably well with the real data when admixture was not too low (above 10%). Other approaches (e.g., Approximate Bayesian Computation or ABC) would probably allow a more thorough investigation of the parameters' space. However, the millions of data sets generated by an ABC approach could anyway not be analyzed by the Structure software for computational reasons. Our aim was mainly to demonstrate that a combined approach using simulated and empirical data can provide some insights into the process of genetic admixture beyond the mere description of admixture patterns.

The simulation parameters were chosen according to the literature and from *in situ* observations made by INRA and fisheries organizations. Other sets of parameters could have been tested including various values of population sizes or straying rates (Jonsson et al. 2003a; Pedersen et al. 2007). In addition, life cycle parameters were kept constant while we know that, for example, populations' sizes or generation time can greatly vary with time (Dumas and Prouzet 2003). The sensitivity analysis revealed that important errors in our estimates of population sizes and/or temporal variations in population sizes may have led to significant biases in admixture estimates. Nevertheless, it seems that this issue mainly influenced admixture estimates in only one population (SIE). We postulated that the increased admixture of wild populations was exclusively attributed to stocking, but we cannot exclude a recent overall increase in straying among wild populations. Indeed, recent studies proposed that dispersal rates could be variable over long time periods (Palstra et al. 2007) and could increase as a result of global change (Valiente et al. 2010). However, if long-distance straying had recently increased, it could not explain the uneven levels of admixture observed in our study populations with strongly stocked populations being highly admixed (COU and SEL) while in weakly stocked rivers admixture is low (SEE) to high (SIE).

To conclude, this study confirms that stocking practices can greatly alter the genetic integrity of managed populations but also the genetic structure among donor and recipient populations. This implies that BMS rivers should be managed independently from other genetic groups to maintain the local genetic diversity and putative local adaptation. From a management perspective, this study allowed estimating the relative performances of stocked and wild individuals and highlighted the role of dispersal as a ‘spreading agent’ of non-native genes into wild salmon populations. This study further demonstrates that the combination of simulation tools and analyses of recent and old archived samples could greatly contribute to the understanding of genetic admixture following the introduction of non-native and/or domestic individuals in wild populations.

## Data Archiving Statement

Data for this study are available in Dryad: DOI: doi:10.5061/dryad.j101c.

## Supporting Information

**Figure S1.** Schematic representation of the metapopulation implemented in Nemo a) before stocking and b) after stocking. Continuous double arrows show dispersal of wild fish among populations.

**Table S1.** Sample sizes, locations, cohorts and abbreviations of the different temporal samples collected in each population.

**Table S2.** Sample size (n), number of alleles per locus (N), allelic richness (AR), F_IS_, expected (H_E_) and observed heterozygosity (H_O_) of the study populations.

**Table S3.** Pairwise F_ST_ with significant values given in bold.
