# Supplementary material for: Understanding admixture patterns in supplemented populations: a case study combining molecular analyses and temporally explicit simulations in Atlantic salmon
Source: Evol Appl. 2012 Jun 14;6(2):218–30. doi: 10.1111/j.1752-4571.2012.00280.x (PMC3689348; doi:10.1111/j.1752-4571.2012.00280.x)
Supplement: Supplementary file 1 [file eva0006-0218-SD1.docx]

Table S1: Sample sizes, locations, cohorts and abbreviations of the different temporal samples collected in each population. Populations used to produce hatchery fish are noticed in italics.

| Population | Coordinates | Cohort | Abbreviation | Sample size |
| --- | --- | --- | --- | --- |
| Sienne | 49.02 : -1.51 | 1985-87 | SIE86 | 40 |
| Sienne | 49.02 : -1.51 | 2002-03 | SIE03 | 37 |
| Sée | 48.68 : -1.38 | 1977-78 | SEE77 | 59 |
| Sée | 48.68 : -1.38 | 1986 | SEE86 | 36 |
| Sée | 48.68 : -1.38 | 2002-03 | SEE03 | 66 |
| Sélune | 48.65 : -1.37 | 1977-78 | SEL77 | 39 |
| Sélune | 48.65 : -1.37 | 1986 | SEL86 | 38 |
| Sélune | 48.65 : -1.37 | 2002-03 | SEL03 | 79 |
| Couesnon | 48.62 : -1.51 | 2002-03 | COU03 | 34 |
| *Aulne* | 48.28 : -4.27 | 1969 | AUL69 | 29 |
| *Aulne* | 48.28 : -4.27 | 2003 | AUL03 | 34 |
| *Gave d'Oloron* | 43.53 : -1.52 | 1984 | GAV84 | 25 |
| *Gave d'Oloron* | 43.53 : -1.52 | 2003 | GAV03 | 29 |

Figure S1: Schematic representation of the metapopulation implemented in Nemo a) before stocking and b) after stocking. Continuous double arrows show dispersal of wild fish among populations. Dashed single arrows show stocking that has occurred during the period studied. Dashed double arrows show dispersal of stocked fish among populations.

Table S2: Sample size (n), number of alleles per locus (N), allelic richness (AR), F_IS_, expected (H_E_) and observed heterozygosity (H_O_) of the study populations. Significant F_IS_ values are given in bold.

|  |  | SIE86 | SIE03 | SEE77 | SEE86 | SEE03 | SEL77 | SEL86 | SEL03 | COU03 | AUL70 | AUL03 | GAV80 | GAV03 | All |
| --- | --- | --- | --- | --- | --- | --- | --- | --- | --- | --- | --- | --- | --- | --- | --- |
|  | n | 40 | 37 | 59 | 36 | 65 | 38 | 38 | 79 | 34 | 29 | 30 | 25 | 29 | 564 |
| BHMS176 | N | 3 | 5 | 5 | 5 | 4 | 5 | 5 | 4 | 4 | 4 | 4 | 4 | 4 | 5 |
|  | A_R_ | 2.5 | 4.0 | 3.1 | 3.5 | 3.5 | 3.2 | 3.5 | 3.6 | 3.9 | 3.3 | 3.9 | 3.9 | 3.8 | 3.7 |
|  | *F*_IS_ | -0.17 | 0.00 | 0.06 | 0.08 | 0.10 | -0.07 | 0.17 | -0.08 | -0.01 | -0.10 | -0.20 | -0.11 | -0.23 |  |
|  | H_E_ | 0.49 | 0.67 | 0.52 | 0.59 | 0.63 | 0.56 | 0.53 | 0.64 | 0.69 | 0.65 | 0.72 | 0.67 | 0.67 |  |
|  | Ho | 0.58 | 0.68 | 0.49 | 0.56 | 0.57 | 0.61 | 0.45 | 0.70 | 0.71 | 0.72 | 0.87 | 0.76 | 0.83 |  |
| BHMS179A | N | 3 | 3 | 2 | 3 | 4 | 2 | 2 | 4 | 3 | 4 | 3 | 5 | 6 | 7 |
|  | A_R_ | 2.2 | 2.4 | 2.0 | 2.3 | 2.5 | 2.0 | 2.0 | 2.5 | 2.8 | 3.2 | 2.9 | 4.2 | 3.8 | 2.7 |
|  | *F*_IS_ | -0.36 | -0.22 | -0.02 | -0.08 | 0.29 | -0.07 | 0.11 | 0.04 | -0.18 | 0.01 | 0.36 | 0.01 | 0.16 |  |
|  | H_E_ | 0.46 | 0.50 | 0.49 | 0.51 | 0.53 | 0.50 | 0.47 | 0.51 | 0.54 | 0.61 | 0.59 | 0.64 | 0.61 |  |
|  | Ho | 0.63 | 0.62 | 0.51 | 0.56 | 0.38 | 0.54 | 0.42 | 0.49 | 0.65 | 0.62 | 0.39 | 0.64 | 0.52 |  |
| BHMS184B | N | 5 | 5 | 2 | 4 | 5 | 3 | 3 | 6 | 4 | 3 | 4 | 6 | 6 | 6 |
|  | A_R_ | 3.0 | 3.3 | 2.0 | 2.5 | 2.6 | 2.6 | 2.2 | 2.8 | 3.8 | 3.0 | 3.5 | 5.2 | 4.8 | 3.4 |
|  | *F*_IS_ | -0.01 | 0.15 | 0.28 | -0.07 | 0.02 | -0.02 | 0.04 | -0.05 | 0.17 | 0.38 | 0.07 | -0.10 | 0.11 |  |
|  | H_E_ | 0.55 | 0.58 | 0.49 | 0.51 | 0.54 | 0.54 | 0.51 | 0.52 | 0.66 | 0.65 | 0.65 | 0.71 | 0.68 |  |
|  | Ho | 0.56 | 0.50 | 0.36 | 0.56 | 0.53 | 0.55 | 0.50 | 0.55 | 0.56 | 0.42 | 0.61 | 0.80 | 0.62 |  |
| BHMS429 | N | 11 | 12 | 10 | 7 | 12 | 12 | 11 | 15 | 12 | 4 | 13 | 9 | 10 | 18 |
|  | A_R_ | 7.0 | 7.7 | 6.6 | 5.7 | 7.0 | 7.8 | 7.5 | 9.1 | 8.3 | 4.0 | 7.7 | 7.1 | 7.8 | 8.4 |
|  | *F*_IS_ | -0.08 | 0.00 | -0.05 | 0.18 | 0.12 | 0.12 | -0.03 | 0.06 | 0.07 | 0.22 | 0.13 | 0.02 | -0.03 |  |
|  | H_E_ | 0.81 | 0.85 | 0.82 | 0.73 | 0.82 | 0.84 | 0.86 | 0.89 | 0.86 | 0.66 | 0.83 | 0.80 | 0.86 |  |
|  | Ho | 0.89 | 0.86 | 0.87 | 0.61 | 0.73 | 0.76 | 0.89 | 0.85 | 0.82 | 0.56 | 0.73 | 0.80 | 0.90 |  |
| SSA85 | N | 11 | 12 | 12 | 12 | 17 | 12 | 11 | 17 | 13 | 9 | 14 | 9 | 13 | 19 |
|  | A_R_ | 7.8 | 7.5 | 8.1 | 7.3 | 8.2 | 8.0 | 6.7 | 8.3 | 8.2 | 6.2 | 8.8 | 7.6 | 8.7 | 8.7 |
|  | *F*_IS_ | 0.12 | -0.04 | 0.09 | -0.02 | 0.08 | 0.02 | 0.05 | 0.00 | -0.04 | 0.11 | -0.07 | -0.05 | 0.03 |  |
|  | H_E_ | 0.85 | 0.82 | 0.86 | 0.81 | 0.85 | 0.84 | 0.79 | 0.86 | 0.84 | 0.79 | 0.86 | 0.86 | 0.87 |  |
|  | Ho | 0.76 | 0.86 | 0.79 | 0.83 | 0.79 | 0.84 | 0.76 | 0.86 | 0.88 | 0.71 | 0.93 | 0.92 | 0.86 |  |
| SSA65 | N | 14 | 14 | 14 | 12 | 14 | 12 | 10 | 15 | 13 | 9 | 13 | 9 | 12 | 19 |
|  | A_R_ | 8.8 | 8.5 | 8.4 | 8.0 | 8.4 | 8.2 | 8.0 | 9.1 | 8.8 | 6.0 | 8.0 | 6.8 | 7.7 | 9.0 |
|  | *F*_IS_ | 0.13 | -0.01 | 0.00 | 0.09 | 0.05 | 0.14 | -0.07 | 0.03 | 0.05 | -0.14 | 0.05 | -0.11 | -0.02 |  |
|  | H_E_ | 0.87 | 0.87 | 0.88 | 0.87 | 0.87 | 0.87 | 0.87 | 0.90 | 0.88 | 0.73 | 0.80 | 0.81 | 0.83 |  |
|  | Ho | 0.76 | 0.89 | 0.88 | 0.81 | 0.84 | 0.76 | 0.95 | 0.87 | 0.85 | 0.84 | 0.77 | 0.92 | 0.86 |  |
| SSOSL85 | N | 14 | 13 | 16 | 11 | 13 | 11 | 12 | 17 | 15 | 10 | 13 | 7 | 10 | 23 |
|  | A_R_ | 8.6 | 8.2 | 8.5 | 8.1 | 8.0 | 7.8 | 8.1 | 8.8 | 9.4 | 6.4 | 7.4 | 6.3 | 7.1 | 8.5 |
|  | *F*_IS_ | -0.01 | -0.05 | 0.15 | 0.04 | 0.03 | 0.10 | 0.01 | -0.01 | 0.03 | 0.12 | 0.05 | 0.11 | 0.14 |  |
|  | H_E_ | 0.86 | 0.86 | 0.86 | 0.87 | 0.87 | 0.85 | 0.86 | 0.88 | 0.89 | 0.69 | 0.82 | 0.79 | 0.82 |  |
|  | Ho | 0.88 | 0.92 | 0.74 | 0.85 | 0.85 | 0.78 | 0.87 | 0.89 | 0.87 | 0.62 | 0.79 | 0.72 | 0.72 |  |
| SSA9 | N | 19 | 18 | 14 | 14 | 16 | 15 | 14 | 19 | 14 | 19 | 18 | 11 | 18 | 26 |
|  | A_R_ | 10.1 | 9.7 | 8.7 | 9.6 | 9.0 | 9.7 | 10.2 | 10.0 | 8.9 | 10.9 | 10.6 | 8.7 | 10.5 | 10.7 |
|  | *F*_IS_ | -0.02 | -0.05 | -0.02 | **-0.14** | -0.05 | 0.02 | 0.08 | 0.04 | 0.07 | -0.04 | -0.05 | 0.07 | 0.05 |  |
|  | H_E_ | 0.89 | 0.89 | 0.88 | 0.86 | 0.88 | 0.89 | 0.90 | 0.90 | 0.87 | 0.91 | 0.91 | 0.68 | 0.89 |  |
|  | Ho | 0.92 | 0.95 | 0.90 | 1.00 | 0.92 | 0.89 | 0.85 | 0.87 | 0.82 | 0.97 | 0.97 | 0.67 | 0.86 |  |
| BHMS235 | N | 15 | 17 | 17 | 15 | 19 | 17 | 14 | 20 | 14 | 9 | 17 | 13 | 16 | 27 |
|  | A_R_ | 9.0 | 9.6 | 9.5 | 9.1 | 9.5 | 9.5 | 9.0 | 10.6 | 8.8 | 6.3 | 10.1 | 8.8 | 10.3 | 10.3 |
|  | *F*_IS_ | -0.10 | -0.01 | -0.03 | 0.02 | 0.00 | -0.08 | 0.00 | 0.03 | -0.01 | -0.13 | -0.03 | 0.19 | 0.10 |  |
|  | H_E_ | 0.88 | 0.89 | 0.90 | 0.87 | 0.89 | 0.89 | 0.88 | 0.92 | 0.86 | 0.78 | 0.90 | 0.87 | 0.90 |  |
|  | Ho | 0.97 | 0.92 | 0.93 | 0.86 | 0.89 | 0.97 | 0.89 | 0.90 | 0.88 | 0.90 | 0.94 | 0.72 | 0.83 |  |
| BHMS217 | N | 7 | 8 | 7 | 6 | 8 | 7 | 7 | 8 | 7 | 6 | 9 | 6 | 8 | 9 |
|  | A_R_ | 5.1 | 6.1 | 5.2 | 5.1 | 5.4 | 5.2 | 5.6 | 5.5 | 6.1 | 4.6 | 5.9 | 4.4 | 5.1 | 5.6 |
|  | *F*_IS_ | -0.13 | 0.17 | 0.07 | 0.05 | 0.12 | -0.07 | -0.11 | 0.12 | 0.00 | 0.19 | -0.07 | 0.00 | 0.00 |  |
|  | H_E_ | 0.71 | 0.81 | 0.76 | 0.77 | 0.78 | 0.73 | 0.77 | 0.78 | 0.83 | 0.75 | 0.79 | 0.55 | 0.61 |  |
|  | Ho | 0.82 | 0.69 | 0.72 | 0.74 | 0.70 | 0.80 | 0.86 | 0.70 | 0.84 | 0.62 | 0.85 | 0.56 | 0.62 |  |
| BHMS111 | N | 9 | 7 | 8 | 8 | 8 | 8 | 8 | 9 | 7 | 6 | 6 | 6 | 6 | 10 |
|  | A_R_ | 6.2 | 5.4 | 5.8 | 5.9 | 5.3 | 5.2 | 5.9 | 5.1 | 4.9 | 4.7 | 4.8 | 5.3 | 4.9 | 5.6 |
|  | *F*_IS_ | -0.11 | 0.21 | -0.04 | -0.06 | -0.11 | -0.08 | -0.02 | -0.07 | -0.03 | -0.03 | 0.00 | 0.00 | 0.25 |  |
|  | H_E_ | 0.75 | 0.67 | 0.75 | 0.74 | 0.71 | 0.68 | 0.76 | 0.71 | 0.70 | 0.69 | 0.73 | 0.79 | 0.76 |  |
|  | Ho | 0.85 | 0.54 | 0.78 | 0.80 | 0.79 | 0.74 | 0.79 | 0.76 | 0.74 | 0.72 | 0.74 | 0.80 | 0.59 |  |
| SSA197 | N | 8 | 13 | 9 | 9 | 8 | 8 | 10 | 15 | 11 | 12 | 12 | 9 | 15 | 25 |
|  | A_R_ | 5.7 | 7.3 | 5.1 | 6.3 | 5.5 | 5.9 | 6.3 | 6.8 | 6.8 | 8.0 | 7.2 | 7.6 | 9.3 | 7.1 |
|  | *F*_IS_ | -0.04 | 0.03 | -0.11 | 0.09 | -0.12 | 0.04 | 0.14 | 0.04 | 0.07 | 0.01 | -0.04 | 0.00 | -0.03 |  |
|  | H_E_ | 0.78 | 0.82 | 0.75 | 0.80 | 0.78 | 0.78 | 0.81 | 0.81 | 0.81 | 0.82 | 0.81 | 0.86 | 0.89 |  |
|  | Ho | 0.82 | 0.81 | 0.83 | 0.74 | 0.88 | 0.76 | 0.71 | 0.78 | 0.76 | 0.83 | 0.85 | 0.88 | 0.93 |  |
| SSA171 | N | 11 | 15 | 12 | 7 | 11 | 6 | 8 | 14 | 11 | 7 | 15 | 9 | 13 | 25 |
|  | A_R_ | 6.7 | 8.5 | 6.6 | 5.3 | 5.9 | 5.2 | 6.0 | 7.2 | 7.0 | 6.5 | 9.7 | 6.8 | 10.4 | 7.8 |
|  | *F*_IS_ | 0.03 | -0.01 | 0.16 | -0.08 | 0.01 | -0.04 | -0.06 | 0.05 | 0.04 | 0.17 | 0.06 | 0.11 | -0.01 |  |
|  | H_E_ | 0.82 | 0.87 | 0.82 | 0.78 | 0.80 | 0.76 | 0.81 | 0.83 | 0.79 | 0.82 | 0.88 | 0.75 | 0.90 |  |
|  | Ho | 0.81 | 0.89 | 0.70 | 0.86 | 0.80 | 0.80 | 0.86 | 0.79 | 0.77 | 0.71 | 0.85 | 0.68 | 0.93 |  |
| BHMS377 | N | 15 | 22 | 20 | 15 | 19 | 17 | 15 | 22 | 18 | 13 | 15 | 13 | 21 | 33 |
|  | A_R_ | 8.7 | 12.0 | 10.2 | 9.4 | 9.7 | 9.5 | 9.5 | 10.7 | 10.5 | 8.2 | 9.7 | 8.4 | 10.8 | 11.2 |
|  | *F*_IS_ | 0.06 | **-0.06** | **-0.09** | 0.01 | 0.02 | -0.02 | 0.04 | -0.03 | 0.01 | -0.01 | 0.16 | 0.06 | 0.13 |  |
|  | H_E_ | 0.85 | 0.93 | 0.91 | 0.89 | 0.89 | 0.89 | 0.89 | 0.92 | 0.91 | 0.84 | 0.90 | 0.84 | 0.89 |  |
|  | Ho | 0.81 | 1.00 | 1.00 | 0.89 | 0.88 | 0.92 | 0.86 | 0.95 | 0.91 | 0.86 | 0.77 | 0.81 | 0.79 |  |
| SSSP2216 | N | 11 | 12 | 17 | 10 | 13 | 13 | 11 | 13 | 12 | 10 | 11 | 12 | 15 | 22 |
|  | A_R_ | 7.0 | 8.0 | 8.8 | 7.3 | 7.3 | 7.6 | 7.1 | 7.8 | 8.0 | 7.3 | 8.1 | 8.5 | 9.6 | 8.3 |
|  | *F*_IS_ | -0.08 | -0.01 | 0.12 | 0.01 | -0.06 | 0.16 | 0.13 | -0.05 | -0.02 | 0.22 | 0.02 | -0.07 | -0.02 |  |
|  | H_E_ | 0.78 | 0.85 | 0.86 | 0.83 | 0.79 | 0.78 | 0.78 | 0.84 | 0.85 | 0.83 | 0.86 | 0.87 | 0.89 |  |
|  | Ho | 0.86 | 0.86 | 0.76 | 0.83 | 0.84 | 0.67 | 0.69 | 0.88 | 0.88 | 0.66 | 0.86 | 0.95 | 0.93 |  |
| BHMS365 | N | 11 | 14 | 15 | 12 | 19 | 12 | 12 | 22 | 18 | 6 | 17 | 13 | 18 | 33 |
|  | A_R_ | 6.6 | 9.3 | 7.8 | 7.8 | 7.6 | 7.6 | 7.6 | 8.9 | 9.6 | 5.1 | 11.2 | 10.2 | 11.1 | 9.2 |
|  | *F*_IS_ | 0.01 | 0.05 | 0.07 | 0.07 | -0.03 | 0.05 | 0.07 | 0.03 | 0.09 | 0.17 | **-0.07** | **-0.08** | **0.34** |  |
|  | H_E_ | 0.82 | 0.89 | 0.86 | 0.84 | 0.85 | 0.84 | 0.84 | 0.88 | 0.89 | 0.71 | 0.92 | 0.90 | 0.91 |  |
|  | Ho | 0.83 | 0.86 | 0.81 | 0.79 | 0.88 | 0.81 | 0.79 | 0.86 | 0.82 | 0.62 | 1.00 | 1.00 | 0.62 |  |
| SSA224 | N | 6 | 7 | 7 | 6 | 5 | 6 | 5 | 8 | 6 | 6 | 4 | 6 | 6 | 9 |
|  | A_R_ | 4.5 | 5.0 | 4.5 | 4.4 | 4.2 | 4.0 | 4.0 | 4.9 | 4.8 | 4.4 | 3.8 | 5.0 | 4.3 | 4.7 |
|  | *F*_IS_ | 0.07 | -0.13 | -0.12 | -0.11 | -0.03 | -0.06 | -0.08 | -0.11 | -0.22 | -0.30 | -0.18 | -0.08 | -0.03 |  |
|  | H_E_ | 0.56 | 0.63 | 0.61 | 0.54 | 0.61 | 0.48 | 0.64 | 0.65 | 0.67 | 0.63 | 0.66 | 0.68 | 0.59 |  |
|  | Ho | 0.53 | 0.72 | 0.69 | 0.60 | 0.63 | 0.51 | 0.70 | 0.72 | 0.82 | 0.83 | 0.79 | 0.75 | 0.62 |  |
| All loci | *F*_IS_ | -0.03 | 0.00 | 0.03 | 0.01 | 0.02 | 0.02 | 0.02 | 0.01 | 0.01 | 0.05 | 0.01 | 0.00 | 0.06 |  |
|  | H_E_ | 0.75 | 0.79 | 0.76 | 0.75 | 0.77 | 0.75 | 0.76 | 0.79 | 0.80 | 0.74 | 0.80 | 0.77 | 0.80 |  |
|  | Ho | 0.78 | 0.80 | 0.75 | 0.76 | 0.76 | 0.75 | 0.76 | 0.79 | 0.80 | 0.72 | 0.81 | 0.79 | 0.77 |  |
|  | m(A_R_) | 6.45 | 7.21 | 6.52 | 6.33 | 6.45 | 6.41 | 6.44 | 7.16 | 7.10 | 5.77 | 7.27 | 6.76 | 7.65 |  |

Table S3: Pairwise F_ST_ with significant values given in bold.

|  | SIE03 | SEE77 | SEE86 | SEE03 | SEL77 | SEL86 | SEL03 | COU03 | AUL69 | AUL03 | GAV84 | GAV03 |
| --- | --- | --- | --- | --- | --- | --- | --- | --- | --- | --- | --- | --- |
| SIE86 | **0.011** | 0.003 | 0.003 | 0.003 | 0.003 | 0.004 | **0.008** | **0.028** | **0.068** | **0.052** | **0.096** | **0.075** |
| SIE03 |  | **0.008** | **0.009** | 0.005 | 0.007 | **0.008** | 0.003 | **0.010** | **0.053** | **0.028** | **0.068** | **0.045** |
| SEE77 |  |  | 0.009 | 0.005 | 0.004 | 0.003 | **0.008** | **0.026** | **0.067** | **0.048** | **0.087** | **0.065** |
| SEE86 |  |  |  | 0.002 | 0.007 | 0.008 | **0.008** | **0.021** | **0.075** | **0.045** | **0.095** | **0.071** |
| SEE03 |  |  |  |  | 0.003 | **0.006** | 0.003 | **0.021** | **0.064** | **0.041** | **0.088** | **0.067** |
| SEL77 |  |  |  |  |  | 0.006 | 0.004 | **0.025** | **0.070** | **0.049** | **0.094** | **0.067** |
| SEL86 |  |  |  |  |  |  | **0.005** | **0.030** | **0.067** | **0.046** | **0.087** | **0.063** |
| SEL03 |  |  |  |  |  |  |  | **0.015** | **0.057** | **0.028** | **0.073** | **0.051** |
| COU03 |  |  |  |  |  |  |  |  | **0.039** | **0.013** | **0.074** | **0.052** |
| AUL69 |  |  |  |  |  |  |  |  |  | **0.025** | **0.079** | **0.073** |
| AUL03 |  |  |  |  |  |  |  |  |  |  | **0.065** | **0.046** |
| GAV84 |  |  |  |  |  |  |  |  |  |  |  | 0.011 |
